# PHT427 as an effective New Delhi metallo-β-lactamase-1 (NDM-1) inhibitor restored the susceptibility of meropenem against *Enterobacteriaceae* producing NDM-1

**DOI:** 10.3389/fmicb.2023.1168052

**Published:** 2023-04-17

**Authors:** Xiaohui Li, Qian Wang, Ji Zheng, Yan Guan, Chennan Liu, Jiangxue Han, Sihan Liu, Tianjun Liu, Chunling Xiao, Xiao Wang, Yishuang Liu

**Affiliations:** ^1^National Laboratory for Screening New Microbial Drugs, Institute of Medicinal Biotechnology, Peking Union Medical College and Chinese Academy of Medical Sciences, Beijing, China; ^2^Key Laboratory of Marine Drugs, School of Medicine and Pharmacy, Ministry of Education, Ocean University of China, Qingdao, China; ^3^Institute of Medical Technology, Peking University Health Science Center, Beijing, China; ^4^Department of Clinical Laboratory, Peking University People’s Hospital, Beijing, China; ^5^Beijing Key Laboratory of Drug Resistance Tuberculosis Research, Beijing Chest Hospital, Beijing Tuberculosis and Thoracic Tumor Research Institute, Capital Medical University, Beijing, China

**Keywords:** PHT427, NDM-1 inhibitor, meropenem, bacterial resistance, carbapenemase

## Abstract

**Introduction:**

With the increasingly serious problem of bacterial drug resistance caused by NDM-1, it is an important strategy to find effective inhibitors to assist β-lactam antibiotic treatment against NDM-1 resistant bacteria. In this study, PHT427 (4-dodecyl-*N*-1,3,4-thiadiazol-2-yl-benzenesulfonamide) was identified as a novel NDM-1 inhibitor and restored the susceptibility of meropenem against *Enterobacteriaceae* producing NDM-1.

**Methods:**

We used a high throughput screening model to find NDM-1 inhibitor in the library of small molecular compounds. The interaction between the hit compound PHT427 and NDM-1 was analyzed by fluorescence quenching, surface plasmon resonance (SPR) assay, and molecular docking analysis. The efficacy of the compound in combination with meropenem was evaluated by determining the FICIs of *Escherichia coli* BL21(DE3)/pET30a(+)-*bla*_*N*DM–1_ and *Klebsiella pneumoniae* clinical strain C1928 (producing NDM-1). In addition, the mechanism of the inhibitory effect of PHT427 on NDM-1 was studied by site mutation, SPR, and zinc supplementation assays.

**Results:**

PHT427 was identified as an inhibitor of NDM-1. It could significantly inhibit the activity of NDM-1 with an IC_50_ of 1.42 μmol/L, and restored the susceptibility of meropenem against *E. coli* BL21(DE3)/pET30a(+)-*bla*_*N*DM–1_ and *K. pneumoniae* clinical strain C1928 (producing NDM-1) *in vitro*. The mechanism study indicated that PHT427 could act on the zinc ions at the active site of NDM-1 and the catalytic key amino acid residues simultaneously. The mutation of Asn220 and Gln123 abolished the affinity of NDM-1 by PHT427 *via* SPR assay.

**Discussion:**

This is the first report that PHT427 is a promising lead compound against carbapenem-resistant bacteria and it merits chemical optimization for drug development.

## 1. Introduction

The multi drug resistance (MDR) of bacteria, especially the prevalence and development of multi drug resistant Gram-negative *Enterobacteriaceae*, poses a huge threat to global health and development (Yewale, 2014). According to the Antimicrobial Resistance Review commissioned by the British government, antimicrobial resistance will cause 10 million deaths every year by 2,050 (de Kraker et al., 2016). WHO has announced twelve most important bacteria, nine of which are Gram-negative bacteria, and the three most important are multidrug resistant Gram-negative bacteria, *Acinetobacter baumannii* and *Pseudomonas aeruginosa* (Tacconelli et al., 2018).

β-lactam antibiotics are the most common and effective drugs in the treatment of Gram-negative bacterial infections. The main drug resistance mechanisms include the expression of β-lactamases which has been studied most, the mutation of penicillin binding protein and the active efflux of drugs (Lima et al., 2020). β-lactamase inhibitors are still the most successful antibiotic adjuvants although they have been used for more than 70 years (González-Bello, 2017). According to the differences between protein sequences, Ambler divided β-lactamases into four types: Ambler A, Ambler B, Ambler C, and Ambler D. The active center of class A, C, and D enzymes have serine residues, also known as serine β-lactamases (SBLs). While the active center of class B enzymes are metal ions (mainly zinc ions), called metallo-β-lactamases (MBLs) (Ambler, 1980). Based on sequence similarity, MBL is divided into three subcategories: B1, B2, and B3 (Garau et al., 2004). B1 subclasses are the most important class of MBLs in clinic, including New Delhi metallo-β-lactamase 1 (NDM-1), which poses a serious threat to human health due to its complex substrate, broad-spectrum function, transferability, etc., (Walsh et al., 2011).

NDM-1 has spread in more than 70 countries worldwide since it was first found in *Klebsiella pneumoniae* isolated from hospitalized patients in 2008 (Yong et al., 2009; Dortet et al., 2014). NDM-1 can hydrolyze almost all β-lactam antibiotics, including carbapenem antibiotics (except aztreonam), therefore it has attracted much attention (Tooke et al., 2019). NDM-1 mainly exists in *K. pneumoniae* and *Escherichia coli* (Poirel et al., 2010). *Enterobacteriaceae* producing NDM-1 was reported in hospital and community acquired infections, including urinary tract infection, sepsis, pulmonary infection, peritonitis, etc., (Poirel et al., 2014). The carbapenem resistant classical *K. pneumoniae* strain carrying *bla*_*NDM*_ was first detected in southwest Iran (Saki et al., 2022). In addition, an NDM-1-producing ST25 *K. pneumoniae* strain was isolated from a 9-day old female newborn diagnosed with intracranial infection in China (Zhao et al., 2022). At present, more than 500 NDM-1 inhibitors have been reported, but there is no clinically available NDM-1 inhibitor except the combination of bicyclic borate inhibitor VNRX-5133 and cefepime which has entered the phase III clinical trial (Abdelraouf et al., 2020). Therefore, it is urgent to find an effective drug that inhibits the resistance of NDM-1.

PHT427 was originally designed as an AKT (protein kinase B) inhibitor (Moses et al., 2009), and could also inhibit PtdIns-dependent protein kinase 1 (PDPK1) (Meuillet et al., 2010). However, the effect on Gram-negative bacteria has not been reported. In this study, PHT427 (4-dodecyl-*N*-1,3,4-thiadiazol-2-yl-benzenesulfonamide) was identified as a novel NDM-1 inhibitor through our high-throughput screening model (Jiangxue, 2019). Notably, the combination of PHT427 with meropenem could attenuate meropenem resistance in NDM-1-producing *E. coli*. Fluorescence quenching and SPR assays suggested that PHT427 was able to bind to NDM-1. Asn220 and Gln123 in the active site played a key role in maintaining the stability of PHT427 and NDM-1 binding. Zinc supplementation assay demonstrated that PHT427 exerted its inhibitory activity by chelating zinc ions at the active site of NDM-1 enzyme. In conclusion, our results indicate that PHT427 is a promising anti-Gram-negative bacteria agent which targets NDM-1.

## 2. Materials and methods

### 2.1. Bacterial strains and chemicals

The engineered strain *E. coli* BL21(DE3)/pET30a(+)-*bla*_*NDM–1*_ with the *bla*_*NDM–1*_ gene (GenBank: AB614355.1) was provided by Professor Xuefu You of the Institute of Medicinal Biotechnology, Chinese Academy of medical sciences (IMB, CAMS). *K. pneumoniae* clinical strains C1928 (producing NDM-1) and C2315 (producing NDM-1 and KPC-2) were supplied by Professor Hui Wang of the Institute of Clinical Laboratory, Peking University People’s Hospital. PHT427 was purchased from Shanghai Pottery Biotechnology Co., Ltd. All antibiotics were obtained from the National Institutes for Food and Drug Control (Beijing, China). The Vero cells and HepG2 cells were presented by Professor Yuhuan Li and Qiyang He (IMB, CAMS), respectively. EDTA was purchased by Sangon Biotech (Shanghai) Co., Ltd. ZnCl_2_ was obtained from J&K Scientific Company. The library of small molecular compounds which contains 13,250 compounds with known biological activities was purchased from Topscience (Shanghai), and the article number was L4000.

### 2.2. Construction of plasmid

The mutations in *bla*_*NDM–1*_ were generated by site-directed mutagenesis using Fast Mutagenesis System (TransGen Biotech, Beijing, China) according to kit instructions as previous described (Li et al., 2017). Key amino acids in NDM-1 were changed to alanines accordingly: Q123A (Gln123 to Ala), D124A (Asp124 to Ala), and N220A (Asn220 to Ala). Positive mutated plasmids were confirmed by DNA Sequencing.

### 2.3. Expression and purification of NDM-1 wild type and mutated enzyme

The pET30a(+)-*bla*_*NDM–1*_ and mutated plasmids were transformed into *E. coli* BL21(DE3) competent cells, respectively. The single clone was cultured in LB medium with 50 μg/mL kanamycin at 37°C. NDM-1 was expressed by the addition of 0.5 mM IPTG and cultured at 22°C for 21 h when the absorbance at 600 nm reach the range of 0.6–0.8. The harvested bacterial cells were disrupted by pressure and centrifuged at 12,000 rpm for 30 min. The proteins which present in the supernatant were purified through Ni^2+^ ion-affinity chromatography using a linear gradient 45–400 mM imidazole in washing buffer (20 mM Tris–HCl, 500 mM NaCl, pH 7.9). The eluted fractions were analyzed by SDS-PAGE followed by Coomassie Blue staining. The protein concentration was measured using Easy II Protein Quantitative Kit (BCA) (TransGen Biotech, Beijing, China), and the purified proteins were stored at −80°C with 50% glycerol.

### 2.4. NDM-1 inhibitor screening

Nineteen compounds were screened at a final concentration of 20 μmol/L through our previous high-throughput screening model of NDM-1 inhibitors (Jiangxue, 2019). Briefly, the assays were carried out in the 96-well plates containing the following ingredients: 10 mmol/L HEPES (pH 7.5), 2.8 U NDM-1 and 62.5 μmol/L meropenem. The EDTA (20 μmol/L) was used as a positive control, and the negative control group only contained DMSO. The inhibition rate was calculated as follows:


$$\%\mathrm{inhibition}=\left(1-\frac{\text{A}\,p-\text{A}\,s}{\text{A}\,p-\text{A}\,n}\right)\times 100\%$$

in which, *Ap* and *An* represented the average absorbance of positive and negative controls, respectively, and *As* was the absorbance of sample. Compounds were perceived as hits when the 80% inhibition limit was achieved.

### 2.5. Enzyme inhibition assay

NDM-1 was incubated with a gradient concentration of PHT427 in 10 mmol/L HEPES (pH 7.5) at 37°C for 15 min. EDTA was used as the parallel positive control and DMSO was used as the parallel negative control. Then the substrate meropenem (final concentration of 60 μmol/L) was added to initiate the reaction and the absorbance at OD_300_ nm which could reflect the activity of NDM-1 was recorded using Enspire 2300 multilabel reader (PerkinElmer) at 37°C. The IC_50_ value was analyzed using GraphPad Prism 8.0.

For the test of Q123A, D124A, and N220A, the enzyme inhibition assay briefly goes as follow: NDM-1, Q123A, D124A, and N220A (final concentration of 0.25 μg/mL) were incubated at 37°C for 15 min in the absence of PHT427, respectively. Other procedures were kept strictly the same.

### 2.6. Fluorescence quenching assay

Fluorescence detection assay was carried out in the 96-well black plate. NDM-1 (final concentration of about 416 μg/mL) was incubated at 37°C for 15 min in the absence or presence of a gradient concentration of PHT427 (31.25–500 μM). The excitation wavelength was 270 nm, and emission spectra were acquired by scanning from 310 to 490 nm using Enspire 2300 multilabel reader (PerkinElmer).

### 2.7. Surface plasmon resonance (SPR) assay

Surface plasmon resonance was performed using Reichert 2SPR with CM5 chip (Reichert, New York, USA). NDM-1, N220A, and Q123A were diluted to 100 μg/ml at 10 mmol/L sodium acetate buffer at pH 4.5 and then immobilized to a CM5 chip by amine coupling, respectively. PHT427 was dissolved in running buffer (PBST containing 1% DMSO) to different concentration gradients (6.25–50 μM) and then injected into the surface of the protein-coupled chip channels at the flow rate of 25 μL/min. The binding affinity was calculated using TraceDrawer software (Reichert, New York, USA).

### 2.8. Molecular docking

The crystal structure of NDM-1 solved at a 2.40 Å resolution was derived from the Protein Data Bank (PBD: 4RBS), which is a complex of NDM-1 and meropenem. PHT427 molecular structure was optimized and then docked with the active pocket of NDM-1 using Discovery Studio 2018 software. The docking model was selected with the highest score to analyze the interaction between NDM-1 and PHT427.

### 2.9. Zinc supplementation assay

The active center of NDM-1 contains two zinc ions, which are necessary for NDM-1 to exert antibiotic hydrolytic activity. In order to investigate whether the inhibition of NDM-1 activity by PHT427 was related to zinc ions, zinc supplementation assay which could test the inhibitory activity of inhibitors against NDM-1 in the presence of Zn^2+^ ions was performed. NDM-1 (final concentration of 0.25 μg/mL) was incubated with PHT427 (20 μM) at 37°C for 15 min in the absence or presence of Zn^2+^ ions (20 μM). EDTA was used as positive control and DMSO was the negative control. Then the substrate meropenem (final concentration of 60 μmol/L) was added to initiate the reaction, and the change in absorbance at 300 nm was monitored on a Enspire 2300 multilabel reader (PerkinElmer) at 37°C for calculation of the inhibition rates.

### 2.10. Determination of minimum inhibitory concentration (MIC) and checkerboard microdilution assays

The MICs of PHT427 in combination with β-lactam antibiotics to *E. coli* BL21(DE3)/pET30a(+)-*bla*_*NDM–1*_, *K. pneumoniae* clinical strains C1928 and C2315 were performed using the broth microdilution method according to the Clinical Laboratory Standards Institute (CLSI)^1^ guidelines. The bacterial cells were cultured at 37°C for about 21 h and the results were observed. For checkerboard microdilution assay, meropenem (0.03125-256 μg/mL) was tested in combination with PHT427 (0-400 μmol/L) against *E. coli* BL21(DE3)/pET30a(+)-*bla*_*NDM–1*_, *K. pneumoniae* clinical strains C1928 (producing NDM-1) and C2315 (producing NDM-1 and KPC-2), in triplicate. Other procedures were kept strictly the same. The fractional inhibitory concentration index (FICI) was determined according to the following equation: FICI = FICA + FICB = CA/MICA + CB/MICB, where MICA and MICB are the MIC values of compounds A and B, respectively, when functioning alone, and CA and CB are the concentrations of compounds A and B at the effective combinations. Synergism was defined when FICI ≤ 0.5, indifference was defined when FICI > 0.5 and < 4, and antagonism was defined when FICI ≥ 4 (Mathers, 2015). A volume of 624.98 μmol/L was set as the MIC of PHT427 against *E. coli* BL21(DE3)/pET30a(+)-*bla*_*NDM–1*_ for the determination of FIC values, and 400 μmol/L was set as the MIC of PHT427 against *K. pneumoniae* clinical strains C1928 (producing NDM-1) and C2315 (producing NDM-1 and KPC-2) for the determination of FIC values.

### 2.11. Cell cytotoxicity assay

Cell Counting Kit-8 (CCK8) was used to test cell cytotoxicity of the compound. Vero cells (the kidney cells of the African green monkey) and HepG2 cells (human hepatocellular carcinoma) were seeded into 96-well culture plates at the density of 2 × 10^3^ cells per well and 5 × 10^3^ cells per well, respectively. The cells were then treated with varying concentrations of PHT427 (0–200 μM). After 48 h, 20 μL of CCK8 reagent was added to each well and then cultured for 1–2 h. Absorbance was measured at 450 nm using Enspire 2300 multilabel reader (PerkinElmer) using wells without cells as blanks, and the IC_50_ values were calculated using GraphPad prism 8.0. All experiments were performed in triplicate.

### 2.12. Acute toxicity assay *in vivo*

Kunming male mice (25 g) were purchased from Beijing Vital River Laboratory Animal Technology (Beijing, China). Experimental mice were randomized to cages of 6 per group for this experiment. The mice were divided into three groups, including Control group (0.5% CMC-Na), PHT427 (L) (500 mg/kg in 0.5% CMC-Na), and PHT427 (H) (1,000 mg/kg in 0.5% CMC-Na). Mice administered via gastric gavage and closely monitored over 72 h. The number of surviving mice was recorded. All animal experimental procedures were performed under the regulations of the Institutional Animal Care and Use Committee of the Institute of Medicinal Biotechnology.

### 2.13. Statistical analysis

All the data were performed on three independent experiments, using GraphPad prism 8.0. The statistical analysis of the results was performed using two tailed *t*-test with ^**^ indicating *P* < 0.01, ^***^ indicating *P* < 0.001, and ^****^ indicating *P* < 0.0001.

## 3. Results

### 3.1. PHT427 inhibits the activity of NDM-1

**FIGURE 1:**
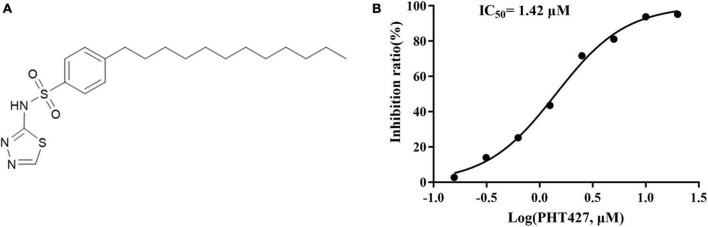
Identification of PHT427 as an effective NDM-1 inhibitor. **(A)** The structure of PHT427. **(B)** Enzyme inhibition assay was used to detect the activity of NDM-1.

NDM-1 inhibitors are screened from the library of small molecular compounds using our previous high-throughput screening model. Nineteen compounds with an inhibition rate of ≥80% were identified. Among them, PHT427 (Figure 1A) showed the best inhibitory activity, which could inhibit NDM-1 in a dose-dependent manner with an IC_50_ of 1.42 μmol/L (Figure 1B). The molecular formula and molecular weight of PHT427 is C_20_H_31_N_3_O_2_S_2_ and 409.61 g/mol, respectively.

### 3.2. The interaction between PHT427 and NDM-1 via fluorescence quenching and SPR assays

**FIGURE 2:**
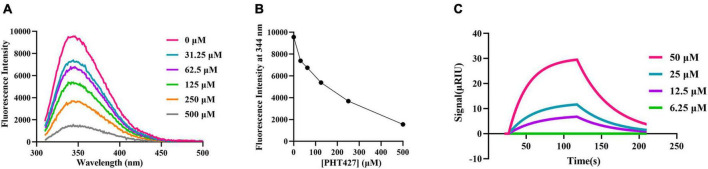
Confirming the interaction between PHT427 and NDM-1. **(A)** Variation of the intrinsic fluorescence spectra of NDM-1. The excitation wavelength was 270 nm, and emission spectra were acquired by scanning from 310 to 490 nm. **(B)** PHT427 quenches the intrinsic fluorescence of NDM-1 in a concentration dependent manner. **(C)** Binding sensorgrams of PHT427. NDM-1 was immobilized to a CM5 chip by amine coupling and the figure was generated by SPR analysis software called TraceDrawer.

The fluorescence quenching and SPR assays were performed to analyze the interaction between PHT427 with NDM-1. We first examined how PHT427 affected the intrinsic tryptophan fluorescence of the NDM-1 enzyme via the fluorescence quenching assay. As shown in Figure 2A, NDM-1 enzyme alone (0 μM) exhibited remarkable florescence at about 344 nm when excited at 270 nm, which was a property of its aromatic amino acid residues. When PHT427 (31.25–500 μM) was added, the fluorescence intensity of the NDM-1 was quenched gradually in a concentration dependent manner, and the fluorescence intensity almost vanished in the presence of the high concentration of PHT427 (Figure 2B). The results demonstrated that PHT427 was able to bind to NDM-1 by a dose-dependent manner. The aromatic amino acid residues in NDM-1 may undergo a conformational change as a result of this PHT427 binding, which will obstruct the formation of the active center and/or the binding of the substrate. The intrinsic fluorescence of NDM-1 was quenched by the intrinsic fluorescence changes induced by PHT427.

An SPR assay was used for further examining the interaction between PHT427 and NDM-1. CM5 SPR chip was coated with NDM-1 by establishing a covalent bond between amine group of N-terminal amino acid and the carboxyl group of CM5 chip, and then PHT427 with different concentration gradients (6.25–50 μM) were injected into the NDM-1 immobilized chambers. The results showed that PHT427 could bind to NDM-1 with a K_*D*_ value of 6.28 × 10^–5^ M (Figure 2C), which suggested a moderate affinity interaction between PHT427 and NDM-1.

### 3.3. Analysis of the molecular docking results

**FIGURE 3:**
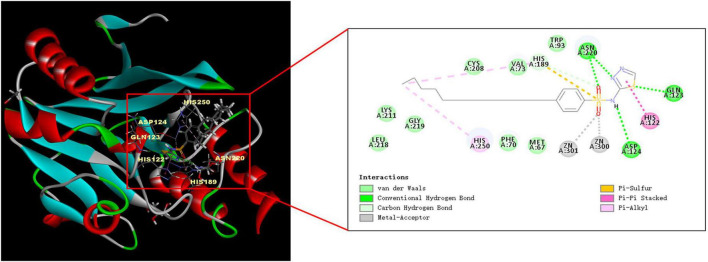
Molecular docking of NDM-1 and PHT427. The active pocket of NDM-1 bound to PHT427.

We used Discovery Studio 2018 software to perform molecular docking between NDM-1 (PBD: 4RBS) and PHT427. As shown in Figure 3, the sulfonamide group of PHT427 is an important functional group for the inhibitory activity of NDM-1. PHT427 acts on Zn1 and Zn2 of NDM-1 through the oxygen atom of the sulfonamide group. The other oxygen atom of the sulfonamide group and the nitrogen atom of the five membered heterocycle form a hydrogen bond with Asn220 to improve the stability of NDM-1 and the compound. Meanwhile, the nitrogen atom of the sulfonamide group forms a hydrogen bond with Asp124, and the sulfur atom of the five membered heterocycle also forms a hydrogen bond with Gln123.

### 3.4. Asn220 mutant and Gln123 mutant are crucial for maintaining the stability of PHT427 and NDM-1 binding

**FIGURE 4:**
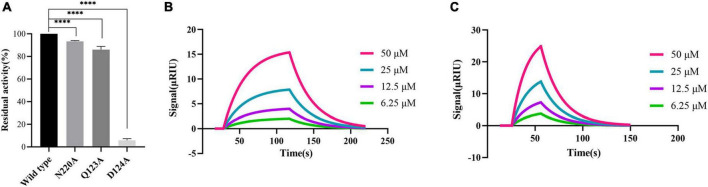
Proving the importance of Asn220 and Gln123 for maintaining the stability of PHT427 and NDM-1 binding. **(A)** Residual activity of wild-type NDM-1, N220A, Q123A and D124A at concentration of 0.25 μg/mL. ^****^ was indicated as *P* < 0.0001. **(B)** Demonstration of the interaction between N220A and PHT427 by SPR, with a K_D_ value of 8.96 × 10^–4^ M. **(C)** Demonstration of the interaction between Q123A and PHT427 by SPR, with a K_D_ value of 1.24 × 10^–4^ M.

According to the molecular docking results, PHT427 forms hydrogen bond interaction with key amino acids Asn220, Asp124, and Gln123. Here, we mutated Gln123, Asp124, and Asn220 to alanines, respectively (as Q123A, D124A, and N220A, respectively). The enzymatic activity of site-directed mutation of Asn220 and Gln123 to alanines (as N220A, Q123A) under identical conditions is 92.29, 86.11%, respectively, indicating that the mutation does not affect the enzymatic activity. While site-directed mutation of Asp124 to an alanine (as D124A) abolished the enzymatic activity by ∼95% under identical conditions, confirming the importance of this residue for the enzymatic activity (Figure 4A). Therefore, N220A and Q123A were used for further research.

To further clarify the importance of Asn220 and Gln123 in maintaining the stability of NDM-1 and PHT427, we subsequently examined the interaction through SPR assay. Compared with NDM-1 and PHT427, the affinity of PHT427 to mutated proteins (N220A, Q123A) were reduced significantly under identical conditions (Figures 4B, C). Hence, these results demonstrate that Asn220 and Gln123 in the active site play a pivotal role on maintaining the stability of PHT427 and NDM-1 binding.

### 3.5. PHT427 exerts its inhibitory activity by chelating zinc ions at the active site of NDM-1 enzyme

**FIGURE 5:**
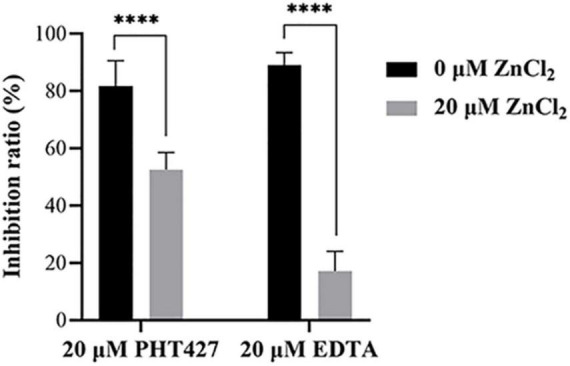
Zinc supplementation assay. Inhibitory activity of NDM-1 enzyme with PHT427, EDTA, PHT427 + ZnCl_2_, and EDTA + ZnCl_2_, respectively. ^****^ was indicated as *P* < 0.0001.

Metal ions are the key cofactor of NDM-1 catalytic function. Zinc ion deficiency causes the inactivation of NDM-1. It has been reported that EDTA exert inhibitory activity only by chelating zinc ions of NDM-1 (Wang et al., 2021). Therefore, a zinc supplementation assay was used to investigate whether PHT427 could exert an inhibitory effect on the NDM-1 enzyme by chelation of zinc ions. As shown in Figure 5, PHT427 (20 μmol/L) and the positive control EDTA (20 μmol/L) exhibited good inhibitory activity against NDM-1 enzyme. However, the addition of zinc ions (20 μmol/L) caused that the inhibitory activity of EDTA to NDM-1 significant decreased about ∼70%, and that of PHT427 to NDM-1 abolished by ∼30% under identical conditions, suggesting that PHT427 could exert its inhibitory activity by chelating zinc ions at the active site of the NDM-1 enzyme. Our results showed that, unlike EDTA, PHT427 may not only exert inhibitory activity by chelating zinc ions of NDM-1, but also through other mechanisms.

### 3.6. PHT427 restores the susceptibility of meropenem against *E. coli* BL21(DE3)/pET30a(+)-*bla*_*NDM–1*_, *K. pneumoniae* clinical strains C1928 (producing NDM-1) and C2315 (producing NDM-1 and KPC-2) *in vitro*

We tested the MICs of β-lactam antibiotics combined with PHT427 at a gradient concentration against *E. coli* BL21(DE3)/pET30a(+)-*bla*_*NDM–1*_. As shown in Table 1, the results showed the MICs of penicillin, ampicillin, ceftazidime, meropenem and biapenem were decreased by 16-, 16-, 8-, 128-, and 16-fold, respectively, in combination with PHT427 at concentration of 39.06 μmol/L.

**TABLE 1:** MIC (μg/mL) of antibiotics against *E.coli* BL21(DE3)/pET30a(+)-*bla*_NDM–1_.

Strain	Antibiotics	MIC (μg/mL)	
		**Alone**	**Combination (Reduction fold)**
*E.coli* BL21(DE3)/pET30a(+)− *bla*_NDM–1_	Penicillin	256	16 (16)
	Ampicillin	>256	16 (16)
	Ceftazidime	256	32 (8)
	Meropenem	32	0.25 (128)
	Biapenem	4	0.25 (16)

PHT427 in combination with antibiotics was tested at a final concentration of 39.06 μmol/L.

**FIGURE 6:**
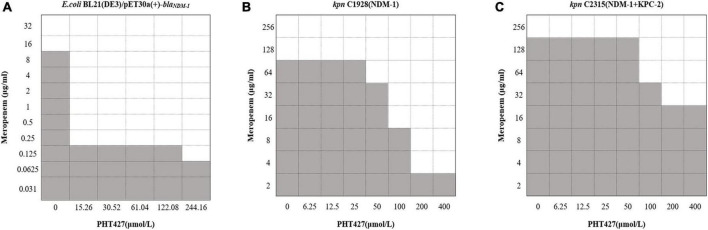
PHT427 restored the susceptibility of meropenem against *E. coli* BL21(DE3)/pET30a(+)-*bla*_NDM–1_, *Klebsiella pneumoniae* clinical strains C1928 (producing NDM-1) and C2315 (producing NDM-1 and KPC-2). Checkerboard microdilution assays for PHT427 in conjunction with meropenem against. **(A)** *E.coli* BL21(DE3)/pET30a(+)-*bla*_NDM–1_. **(B)** *Klebsiella pneumoniae* clinical strain C1928 (producing NDM-1). **(C)** *Klebsiella pneumoniae* clinical strain C2315 (producing NDM-1 and KPC-2).

To verify the above conclusion, we further evaluated the synergistic effect of the combination of PHT427 with meropenem against *E. coli* BL21(DE3)/pET30a(+)-*bla*_*NDM–1*_, *K. pneumoniae* clinical strains C1928 (producing NDM-1) and C2315 (producing NDM-1 and KPC-2) by checkerboard microdilution assays. PHT427 combined with meropenem can reduce the MIC of meropenem (from 16 μg/mL to 0.25 μg/mL) against *E. coli* BL21(DE3)/pET30a(+)-*bla*_*NDM–1*_. The FICI was 0.04, which indicated the synergistic interaction between them (Synergy is defined for FIC index ≤ 0.5) (Figure 6A). In addition, the synergistic effects were also evaluated on *K. pneumoniae* clinical strains C1928 (producing NDM-1) and C2315 (producing NDM-1 and KPC-2), and the FIC index values were 0.38 and 0.50, respectively (Figures 6B, C). In summary, PHT427 could restore the susceptibility of meropenem against *E. coli* BL21(DE3)/pET30a(+)-*bla*_NDM–1_, *K. pneumoniae* clinical strains C1928 (producing NDM-1) and C2315 (producing NDM-1 and KPC-2) *in vitro*.

## 4. Discussion

The emergence and spread of carbapenem resistant *Enterobacteriaceae* (CRE) has posed a great threat to human and animal health (Zhang et al., 2017). NDM-1 has spread rapidly worldwide since its discovery. Food producing animals in China are important hosts of NDM positive *E. coli* (Kuang et al., 2022). The clinical and microbiological data which were collected from 96 ICUs in 78 hospitals in Henan Province, China, showed that the positive rate of carbapenem resistant *Enterobacteriaceae* (CRE) in intensive care units (ICUs) was very high (Guo et al., 2022). In addition, pathogens producing carbapenemase also appeared in coastal waters, and an NDM-1-positive *E. coli* strain belonging to the international clone sequence type (ST) 162 was identified in a pygmy sperm whale (*Kogia breviceps*) (Sellera et al., 2022). Therefore, it is urgent to develop a new antimicrobial agent against carbapenem-resistant bacteria. In the past 20 years, only six new antibiotics have been approved, and all of them are ineffective against Gram-negative bacteria (Butler et al., 2017). Combination therapies, including combination I (antibiotic + antibiotic), combination II (antibiotic + non-antibiotic) and combination III (non-antibiotic + non-antibiotic) offer a promising pipeline for the discovery and development of new anti-infective regimens in the post-antibiotic era (Liu et al., 2021). Non-antibiotic compounds can enhance antibiotic activity by blocking resistance, enhancing intracellular antibiotic accumulation, complementing bactericidal mechanisms, inhibiting signaling and regulatory pathways, or enhancing the host response to bacterial infection (Liu et al., 2019). Combination II (antibiotic + non-antibiotic) has been viewed as a more practical and efficient option than developing novel antibiotics for monotherapy (Olsen, 2015; Czaplewski et al., 2016). Moreover, combination II approach prolongs the life of well-established and clinically validated antibiotics, and significantly shortens development time and cost, while ensuring the safety of drugs (Liu et al., 2021). The outstanding success is inhibitors of β-lactamases, such as amoxicillin-clavulanic acid pair, ceftazidime-avibactam, ceftolozane-tazobactam, meropenem-vaborbactam, imipenem-relebactam etc., (Yahav et al., 2020). Cefepime in combination with VNRX-5133 has shown highly effective antibacterial activity against carbapenem-resistant *Enterobacteriaceae* and *Pseudomonas aeruginosa*, and is currently in a phase III clinical trial (Liu et al., 2019; Vázquez-Ucha et al., 2020).

PHT427 inhibited pleckstrin homology domain targeting AKT and the PtdIns-3-kinase/PtdIns-dependent protein kinase 1 (PDPK1)/Akt (protein kinase B). It was also effective for pancreatic cancer, breast cancer, non-small cell lung cancer (Moses et al., 2009; Meuillet et al., 2010; Miller et al., 2021). Besides, PHT427 had greater additive activity than paclitaxel in breast cancer and erlotinib in non-small cell lung cancer (Meuillet et al., 2010), and improved the treatment of Mia PaCa-2 pancreatic cancer through poly(lactic-co-glycolic acid) (PLGA) nanoparticles (Kobes et al., 2016). Meanwhile, PHT427 has also been reported in the research of microbial system. It was identified as a new antimicrobial agent against *Staphylococcus aureus* causing bovine mastitis, which effectively inhibited the FeoB activity and the growth of Gram-positive food borne bacteria (Shin et al., 2021). However, its inhibitory activity against drug-resistant Gram-negative bacteria has not been reported. In this study, PHT427, a NDM-1 inhibitor, was screened from the library of small molecule compounds based on our previous high-throughput screening model, with an IC_50_ of 1.42 μmol/L. Additionally, we confirmed the interaction between PHT427 and NDM-1 via fluorescence quenching and SPR assays.

The reported NDM-1 inhibitors are mainly divided into three categories according to their interactions with the NDM-1 protein. The first species of inhibitors act on the zinc ion of NDM-1 active site directly, such as Ethylenediamine derivatives. The second type of inhibitors block the binding of NDM-1 to the substrate by acting on the amino acid residues of NDM-1, like Magnolol and its derivatives. The third kind of inhibitors act on the zinc ions at the active site of NDM-1 and the catalytic key amino acid residues at the same time, and they are considered to be the most potential NDM-1 inhibitors, such as captopril and its derivatives (Linciano et al., 2019; Wang et al., 2021). Our molecular docking results showed that PHT427 belonged to the third category of NDM-1 inhibitors. PHT427 acts on Zn1 and Zn2 of NDM-1 through the oxygen atom of the sulfonamide group and forms hydrogen bond interaction with key amino acids Asn220, Asp124, and Gln123. Asp124 participates in the coordination of Zn2, and Zn1 is connected to Zn2 through the side chain of Asp124 (Skagseth et al., 2017; Linciano et al., 2019). Gln123 and Asp124 form hydrogen bond interaction with oxygen atoms adjacent to hydrophobic β-lactam R groups and hydrophilic pores for substrate bonding (Groundwater et al., 2016). Asn220 participates in substrate recognition and hydrolysis, and can stabilize tetrahedral intermediates product by combining with Zn1 to form oxygen anion pore. In addition, Asn220 can form hydrogen bond with carbonyl oxygen of the substrate (Groundwater et al., 2016). We mutated Gln123, Asp124, and Asn220 to alanines, respectively (as Q123A, D124A, and N220A, respectively), and the results showed that N220A and Q123A significantly reduced the interaction compared with NDM-1-wild type. This supported our molecular docking results. Moreover, the mutation of Asp124 reduced the enzymatic activity significantly, which indicated that the importance of this residue for the enzymatic activity. Zinc supplementation assay demonstrated that PHT427 exerted its inhibitory activity by chelating zinc ions at the active site of NDM-1 enzyme. Therefore, the mechanism study showed that PHT427 acts on the zinc ions at the active site of NDM-1 and the catalytic key amino acid residues simultaneously. Additionally, checkerboard microdilution and MIC assays showed that PHT427 was effective for *E. coli* BL21(DE3)/pET30a(+)-*bla*_NDM–1_, and more importantly, it had strong inhibitory activity on *K. pneumoniae* clinical strains C1928 (producing NDM-1) and C2315 (producing NDM-1 and KPC-2). Meanwhile, the combination of PHT427 and meropenem had synergistic effect. However, the synergism effect of PHT427 with meropenem against clinical strains was inferior to the *E. coli* BL21(DE3)/pET30a(+)-*bla*_NDM–1_ isolate. This could be explained by the more complicated drug resistance mechanism of clinical strains, including expression of β-lactamases, the mutation of penicillin binding protein and the active efflux of drugs efflux pumps, etc., (Lima et al., 2020). This phenomenon was consistent with the reported that the synergism effect of 3-Bromopyruvate with β-lactam antibiotics against clinical strains was inferior to the *E. coli* BL21 isolate (Kang et al., 2020). Based on the above results, we believe that PHT427 could be combined with antibiotics to treat carbapenem resistant strains. Furthermore, PHT427 displayed low cytotoxicity with an IC_50_ value of 104.70 ± 3.33 μmol/L for Vero cells, and an IC_50_ value of 76.92 ± 0.57 μmol/L for HepG2 cells (Supplementary Figure 1). *In vivo* acute toxicity assay was performed using male Kunming mice. The surviving number of PHT427 (L) group is 5/6 and that of PHT427 (H) group is 5/6, indicating the safety of the compound in animals. PHT427 showed relatively good safety and does not cause significant changes in body weight and blood biochemistry after oral administration for more than 5 days (Meuillet et al., 2010). Therefore, our results showed that PHT427 is a promising lead compound against carbapenem-resistant bacteria and it merits chemical optimization for drug development. However, we have not evaluated whether PHT427 combined with existing antibiotics can effectively treat carbapenem-resistant bacterial infection *in vivo*, which will be further studied in the future.

## 5. Conclusion

Our research showed that PHT427 was a novel and effective NDM-1 inhibitor. PHT427 could restore the susceptibility of meropenem against *E. coli* BL21(DE3)/pET30a(+)-*bla*_NDM–1_, *K. pneumoniae* clinical strains C1928 (producing NDM-1) and C2315 (producing NDM-1 and KPC-2) *in vitro*. The mechanism study showed that PHT427 acts on the zinc ions at the active site of NDM-1 and the catalytic key amino acid residues simultaneously, and Asn220 and Gln123 at the active site were vital for maintaining the stability of PHT427 and NDM-1 binding.

## Data availability statement

The datasets presented in this article are not readily available because some of these data are needed for follow-up research. Requests to access the datasets should be directed to YL, liuys@imb.pumc.edu.cn.

## Ethics statement

All animal experimental procedures were performed under the regulations of the Institutional Animal Care and Use Committee of the Institute of Medicinal Biotechnology, and Approval Number is IMB-20230310D_3_02.

## Author contributions

XL and QW conducted to the research and wrote the manuscript. YG, CL, SL, TL, and CX helped with the experimental process. JZ performed the checkerboard microdilution assay. XL and JH analyzed the data. XL, XW, and YL revised the manuscript. All authors contributed to the article and approved the submitted version.
