# Supplementary material for: PHT427 as an effective New Delhi metallo-β-lactamase-1 (NDM-1) inhibitor restored the susceptibility of meropenem against Enterobacteriaceae producing NDM-1
Source: Front Microbiol. 2023 Apr 17;14:1168052. doi: 10.3389/fmicb.2023.1168052 (PMC10150926; doi:10.3389/fmicb.2023.1168052)
Supplement: Supplementary file 1 [file Data_Sheet_1.docx]

**Supplemental Figures**

**Figure S1**


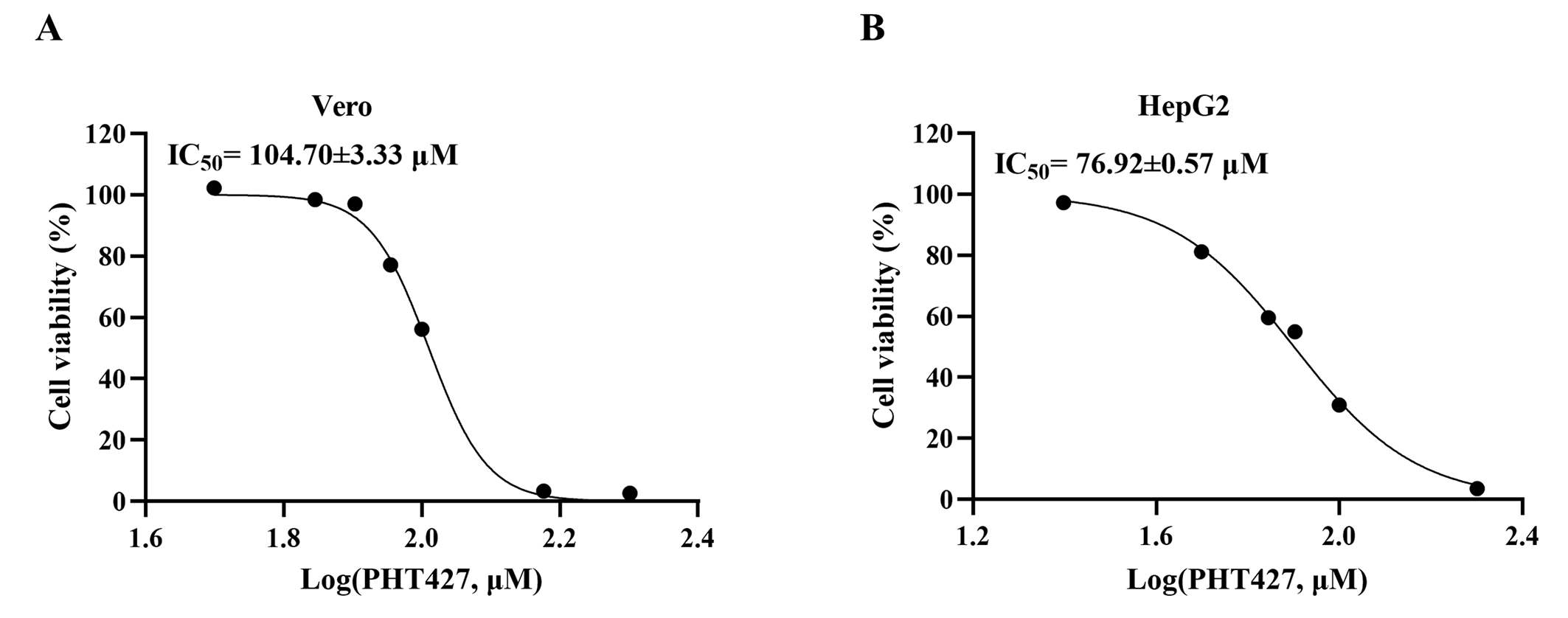


Figure S1 Effects of PHT427 on the viability of different cell types. The cell viabilities of (A) Vero cells (the kidney cells of the African green monkey) and (B) HepG2 cells (human hepatocellular carcinoma) according to Cell Counting Kit-8 cell viability reagent at 48 h culture are shown.
